# Decreased Mitochondrial Function, Biogenesis, and Degradation in Peripheral Blood Mononuclear Cells from Amyotrophic Lateral Sclerosis Patients as a Potential Tool for Biomarker Research

**DOI:** 10.1007/s12035-020-02059-1

**Published:** 2020-08-25

**Authors:** Beatriz Grisolia Araujo, Luiz Felipe Souza e Silva, Jorge Luiz de Barros Torresi, Amanda Siena, Berenice Cataldo Oliveira Valerio, Mariana Dutra Brito, Tatiana Rosado Rosenstock

**Affiliations:** 1 Department of Physiological Science, Santa Casa de São Paulo School of Medical Science, Rua Doutor Cesário Motta Júnior, 61 - Vila Buarque, São Paulo, SP CEP 01221-020 Brazil; 2 Department of Neurology, Irmandade da Santa Casa de Misericórdia de São Paulo, São Paulo, SP Brazil

**Keywords:** Amyotrophic lateral sclerosis, Peripheral blood mononuclear cells, Patients, Mitochondrial depolarization, Mitochondrial fission, Biogenesis

## Abstract

Amyotrophic lateral sclerosis (ALS) is a multifactorial and progressive neurodegenerative disease of unknown etiology. Due to ALS’s unpredictable onset and progression rate, the search for biomarkers that allow the detection and tracking of its development and therapeutic efficacy would be of significant medical value. Considering that alterations of energy supply are one of ALS’s main hallmarks and that a correlation has been established between gene expression in human brain tissue and peripheral blood mononuclear cells (PBMCs), the present work investigates whether changes in mitochondrial function could be used to monitor ALS. To achieve this goal, PBMCs from ALS patients and control subjects were used; blood sampling is a quite non-invasive method and is cost-effective. Different parameters were evaluated, namely cytosolic calcium levels, mitochondrial membrane potential, oxidative stress, and metabolic compounds levels, as well as mitochondrial dynamics and degradation. Altogether, we observed lower mitochondrial calcium uptake/retention, mitochondria depolarization, and redox homeostasis deregulation, in addition to a decrease in critical metabolic genes, a diminishment in mitochondrial biogenesis, and an augmentation in mitochondrial fission and autophagy-related gene expression. All of these changes can contribute to the decreased ATP and pyruvate levels observed in ALS PBMCs. Our data indicate that PBMCs from ALS patients show a significant mitochondrial dysfunction, resembling several findings from ALS’ neural cells/models, which could be exploited as a powerful tool in ALS research. Our findings can also guide future studies on new pharmacological interventions for ALS since assessments of brain samples are challenging and represent a relevant limited strategy.

**Electronic supplementary material:**

The online version of this article (10.1007/s12035-020-02059-1) contains supplementary material, which is available to authorized users.

**Figure:**
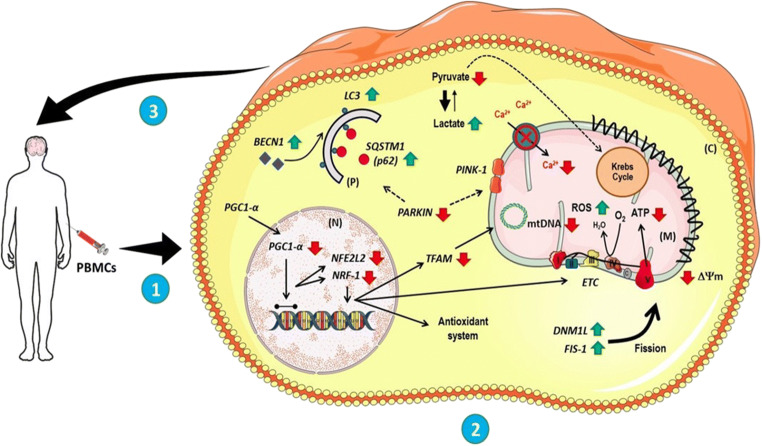
Graphical abstract

## Introduction

Amyotrophic lateral sclerosis (ALS) is a fatal disorder characterized by a specific and progressive degeneration of upper and lower motor neurons followed by muscular atrophy [1–9]. Although several pathological mechanisms have been postulated to explain ALS genesis and progression, this matter is still under debate and goes beyond genetic alterations; mutations (~ 24 genes) account for 68% of familial cases of ALS (fALS) and for just 11% of sporadic ALS (sALS) [10]. In this respect, mitochondrial dysfunction has assumed greater importance, since alteration of energy supply is one of the main features of ALS [11–16]. Nevertheless, the importance of mitochondria is not restricted to adenosine triphosphate (ATP) production [17]; the “metabolic organelle” is also involved in many cellular processes, namely, calcium homeostasis, cellular growth, cellular differentiation, cell death, antioxidant metabolism, and axonal transport [18–21].

Unfortunately, there is no cure or effective therapy for ALS [22], and the diagnosis often occurs in the late stages of the disease and usually accompanies a 50% loss of motor neurons [16]. Therefore, the development of strategies to allow ALS detection and development tracking would be of significant medical value. Toward this end, attention has been focused on potential biological components as biomarkers, which may enable not only a reliable diagnosis but also a predictable follow-up and efficacy assessments of therapeutic interventions [23]. In the last decades, several ALS-related biological biomarkers have been recorded [24–33], some of which are from blood [34–38]. Interestingly, biomarkers from blood samples are receiving increased attention and have even contributed to the evaluation of oxidative stress in ALS patients treated with riluzole (Rilutec®) [39].

Thus, considering that mitochondrial deregulation is associated with ALS and that correlation between gene expression in human brain tissue and peripheral blood mononuclear cells (PBMCs) has already been established [40], the present work investigates whether changes in mitochondrial function could be used to monitor ALS. To achieve this goal, PBMCs from ALS patients and control subjects were used; blood sampling is a fairly non-invasive method and is cost-effective. Different parameters were evaluated in this study, namely, cytosolic calcium levels, mitochondrial membrane potential, oxidative stress, and metabolic compound levels, in addition to gene expression associated with mitochondrial metabolism, dynamics, and degradation.

In PBMCs from ALS patients, we observed an overall lower mitochondrial calcium uptake/retention, mitochondrial depolarization, and redox homeostasis unbalance. Moreover, ALS PBMCs showed a significant decrease in critical metabolic genes and a diminishment in mitochondrial biogenesis and content. These findings seem to occur in parallel to an increase in mitochondrial fission and autophagy-related gene expression, despite a reduction in mitochondrial degradation signaling. Accordingly, ALS cells present a decrease in energy-producing metabolic compounds, such as ATP production and pyruvate, corroborating the fact that mitochondrial deregulation can lead to an energetic deficiency in peripheral cells as an ultimate consequence of the disorder. Thus, mitochondrial function evaluation in PBMCs could be a valuable strategy to detect ALS, as well as to assess its progression and therapy efficacy.

## Material and Methods

### Subjects

The work presented was a hospital-based case-control study, in which patients and control subjects were recruited at the Neurology Department from Irmandade da Santa Casa de Misericórdia de São Paulo (ISCMSP). We got access to 14 ALS individuals. According to El Escorial, clinical diagnosis of ALS individuals was performed based on the presence of clinical signs, which requires upper and lower motor symptoms and a history of progressive disability [41]. At ISCMSP, physicians classified ALS patients in familial when subjects mention that their relatives (first or second degree) are clinically affected—without genetically testing them. Because of the patients’ physical conditions, named for delicate veins, sampling has been carried out in 10–11 affected individuals. Importantly, blood samples were collected in two different time points; the first one (blood sampling 1, BS1) was executed soon after medical evaluation; the second blood sampling (BS2) was collected in 6 patients after 3 months of BS1.

The inclusion criteria of control subjects were (i) no clinical signs of any neuromotor disorder (e.g., frontotemporal dementia, FTD), (ii) no family members with ALS, and (iii) no metabolic disorder. The exclusion criteria were as follows: (i) present any pathology associated to ALS, such as respiratory diseases, difficulty in swallowing, and cognitive disorders, (ii) have comorbidities, namely diabetes and hypertension, (iii) show acute and chronic inflammatory disorders, and (iv) have smoking habits. Control subjects were age- and gender-matched.

### Sampling and Human Peripheral Blood Mononuclear Cell Isolation

Blood from all participants was collected in heparinized tubes (8 mL/each). Following this procedure, blood was transferred to tubes containing Ficoll® (Paque Plus GE Healthcare, 17-1440-02), previously diluted in phosphate buffer (1:1) (PBS, mM: 137 NaCl, 2.7 KCl, 1.8 KH_2_PO_4_, 10 Na_2_HPO_4_, 2H_2_O, pH 7.4). Soon after, samples were centrifuged at 3000 rpm for 20 min (min) at 18 °C. PBMC-containing layer (a cloudy ring) was then transferred to a new centrifuge tube. The serum, located in the top layer of the gradient, was aliquoted (400 μL was maintained at room temperature for further use—in the culture medium). The collected cells were diluted in 45 mL of sterile PBS and centrifuged at 2000 rpm for 10 min at 18 °C. Pellets were cultured, as described below.

### Peripheral Blood Mononuclear Cell Culture

The final pellet containing PBMCs was resuspended in RPMI 1640 medium supplemented with 10% (v/v) serum; each participant’s PBMCs were cultured in its serum. Each cell suspension was transferred to four wells of a six-well plate (2 mL of medium per well). Cells were formerly kept at 37 °C and 5% CO_2_ for 18–24 h [42]. Shortly, all the desired experiments were performed.

### Evaluation of Mitochondrial Functional Parameters

To check mitochondrial functional parameters, different experiments were conducted. To analyze calcium homeostasis, 3 × 10^5^ cells were allocated into a well (in a 96-well plate). At that point, cells were loaded for 1 h at 37 °C with Fluo-4-AM (10 μM), a cytosolic calcium indicator, in microscopy medium (mM: 120 NaCl, 3.5 KCl, 0.4 KH_2_PO_4_, 5 NaCOH_3_, 1.2 NaSO_4_, 20 HEPES, and glucose; pH 7.4) supplemented with 1 mM of calcium chloride (CaCl_2_) in the presence of Pluronic F-127™ (20%), a nonionic detergent which facilitates the fluorescent probe entry into the cells [43–47]. Experiments were performed using the spectrofluorometer Spectramaxi3™ (485-nm excitation; 525-nm emission).

To investigate, indirectly, mitochondrial membrane potential (ΔΨm), cells (3 × 10^5^/well in the 96-well plate) were incubated with tetramethylhydrodamine ethyl ester (TMRE) (500 nM, 1 h) at 37 °C, in microscopy medium [43–46, 48–53]. TMRE is a fluorescent cationic indicator that accumulates preferentially into negative mitochondria (showing mitochondrial polarization). The experiments were also run on the plate reader Spectramaxi3™ (540-nm excitation; 590-nm emission).

The oxidative stress level was evaluated after 3 × 10^5^ cells were transferred to each well of a 96-well plate and incubated with carboxylated diclohydro-fluorescein reagent (CM-H_2_DCF-DA) (20 μM, 1 h, at 37 °C) in microscopy medium [43–46, 48, 53]. CM-H_2_DCF-DA is used to detect the generation of reactive oxygen intermediates and passively diffuse into the cytosol, where it is cleaved by esterases forming DCFH. DCFH is oxidized to the green fluorescent compound 2,7-dichlorofluorescein (DCF). Thus, the higher is the level of reactive oxygen species (ROS) within the cell, the higher is the fluorescence signal. The experiments were also performed on Spectramaxi3™ (495-nm excitation; 520-nm emission).

All experiments were acquired for 10 min. In the first 5 min, the basal fluorescence was measured; after that, another 5 min was recorded to measure fluorocarbonyl cyanide phenylhydrazone (FCCP, 5 μM) effect, a mitochondrial protonophore used as an internal control. The baseline fluorescence was considered the mean of the last 5–8 points of the initial reading (before FCCP), while the FCCP outcome was calculated as the mean of the first 5–8 readings after FCCP [43–46, 48, 53]. All experiments were performed in duplicate, and data were represented as the percentage of the control group.

### Expression of Mitochondrial Function–Related Genes

The expression of genes related to mitochondrial metabolism, dynamic, and degradation was evaluated by real-time PCR (qRT-PCR) [51–53] in 7500 Real-Time PCR Instrument (ThermoFisher Scientific). Briefly, messenger RNA (mRNA) of about 100,000 cells was extracted with Purezol (BioRad) according to the manufacturer’s protocol. The complementary DNA (cDNA) was obtained using 1 μg mRNA, and the iScript DNA Synthesis Kit (Biorad) (total volume of 20 μL). For each qRT-PCR reaction, performed with the SsoAdvanced™ Universal SYBR®Green Supermix kit (BioRad), 200 ng of cDNA and 300 nM of primer (Foward and Reverse) were used. The template was performed with an initial cycle at 95.0 °C for 30 s, followed by 45 cycles of 95 °C (15 s) and 60 °C (30 s).

The expression of the following genes was evaluated (primers sequences are in the Supplementary Material): *Nuclear respiratory factor 1* (*NRF1*) and *Nuclear factor* (*erythroid-derived2*)*-like 2* (*NFE2L2*), to evaluate nuclear-encoded electron transport chain subunits transcription and antioxidant defense; *Peroxisome proliferator-activated receptor gamma coactivator 1-alpha* (*PGC-1α*); *mitochondrial transcription factor A* (*TFAM*) and *tRNAleu*, to investigate mitochondrial biogenesis and content (*tRNAleu* is within an area of mtDNA that is rarely deleted and has few polymorphisms); *Dynamin related protein 1* (*DNM1L*) and *Mitochondrial fission 1* (*FIS-1*), to verify mitochondrial dynamics; *PTEN-induced putative kinase protein 1* (*PINK1*) and *E3 ubiquitin-protein ligase parkin* (*PARKIN*), to investigate mitochondrial degradation signaling; and *Beclin 1* (*BECN1*), *Microtubule-associated proteins 1A/1B light chain 3* (*LC3*) and *Sequestosome-1* (*SQSTM1*), to check changes in autophagy [54–70]. Gene expression was determined with 2^−ΔΔCT^ [71].

### Measurement of Adenine Nucleotides and Metabolic Compounds

Afterward, we evaluated the levels of bioproducts related to energy metabolism, namely ATP, adenosine diphosphate ADP, pyruvate, and lactate. Then, total protein extraction was performed as described previously [46; 50-53]. Briefly, cells were resuspended in Ripa buffer (mM: 20 Tris, 100 NaCl, 2 EDTA, 2 EGTA, 1% Triton X-100, pH 7.0) supplemented with MS-SAFE (protease and phosphatase inhibitor) (Sigma-Aldrich) and were frozen-thaw placed in liquid nitrogen (frozen) and hot water bath (37 °C) for three times. Samples were then sonicated for 3 min in a Sonicator Bath to be then centrifuged for 10 min at 1400 rpm. The final homogenate was considered the total fraction. We quantified protein levels, and we standardize 50 μg to be added in all reactions. All energetic compounds were measured using commercially available kits following (Abcam, ab83355, ab83359, ab65342, ab65330).

### Statistical Analysis

Results are expressed as mean ± standard deviation (SD) of the number of independent experiments indicated in figure legends. The fluorescence was normalized to 1 to assemble graphic lines, and data are represented in relation to the control group (in percentage). Gene expression was determined with 2^−ΔΔCT^ (normalizing values to actin expression in relation to the control group), and results regarding the bioenergetics compounds are also represented concerning the control group (in percentage). The graphs were put together using the GraphPad Prism 6 program (GraphPad Prism Version 6.0), and the same program was used to execute the statistical analysis. We performed one-way ANOVA followed by post hoc Bonferroni and Student’s *t* test to achieve statistical comparisons between groups. It was considered statistically different *p* < 0.05.

## Results

### Subjects Characteristics and Socio-Demographic Data

All outcomes regarding demographic and clinical data are shown in Table 1. Information concerning age, gender, time with the disease, current medication, first motor symptoms, and El Escorial are listed. Our work included 12 control subjects and 14 patients, which were evaluated according to the El Escorial criteria [41]. It is essential to mention that the small sample size was due to several subjects in the Neurology Department presenting motor neuron disorder that was not classified as ALS. In Table 1, we also indicated the familial cases among ALS patients and the presence of other neurodegenerative diseases. Data about age and gender of control subjects are also presented.

**Table 1:** Demographic characteristics of ALS patients and control subject at the time of blood sampling at Irmandade da Santa Casa de Misericórdia de São Paulo (ISCMSP). In total, 12 control individuals and 14 patients were recruited

ALS patients		Male (7)/female (7)		Age (mean), 52	
Control subjects		Male (6)/female (6)		Age (mean), 49	
No.	Age	Duration	Medication	First motor signals	El Escorial
1	63	9 years	Riluzole	Left hemisphere	Definite^1^
2	45	3 years	Riluzole	Right superior	Definite
3	70	3 years	Riluzole	Right superior	Definite^2^
4	56	2 years	Riluzole	Left hemisphere	Definite
5	57	1 year	Riluzole	Left lower	Probable^2^
6	46	12 years	Riluzole	Left lower	Probable
7	33	2 years	Riluzole	Right superior	Definite
8	55	5 years	Riluzole	Lower	Definite
9	44	4 years	Riluzole	Superior	Possible
10	51	7 years	Riluzole	Right superior	Definite^3^
11	59	5 years	Riluzole	Bulbar	Definite
12	56	4 years	Riluzole	Left lower	Definite
13	40	6 years	Riluzole	Bulbar	Probable
14	57	3 years	Riluzole	Right superior	Definite

^1^ALS patient with multiple sclerosis

^2^ALS patients with motor diseases cases in relatives

^3^fALS (familiar case)

Of the 14 ALS subjects in this study, 78.5% of them have presented with the disease for 6 years or less (1–6 years of ALS duration). Only 21.5% of patients have had ALS for 7 years or more (one subject has presented with the disease for 12 years). Despite such a range, the duration of ALS seems not to be correlated with either the diagnosis of fALS or with different outcomes related to mitochondrial functional parameters and gene expression, once the results of those patients were under the group’s mean (see below). Importantly, all individuals evaluated were Caucasian. As mentioned previously [9], we believe that ethnicity is crucial to the interpretation of results, since metabolism can be modulated, at least partially, by polymorphisms that might be ethnically dependent [9].

The mean age of ALS individuals in our study is 52 years, while in the controls is 49 years. Moreover, there are 7 ALS female patients and seven males; the same proportion is found in the control group. Curiously, just one patient has fALS, meaning that 92.86% has sALS; the mother, uncle, and two cousins of the fALS patient are also affected.

Furthermore, none of ALS patients smokes, and the most predominant first motor symptoms are the upper or lower limbs (known as the spinal form)—42.85% of each; just 14.3% of patients presented bulbar symptoms first (Table 1). The treatment used by all patients of this cohort is riluzole, the most used medication in ALS [72].

### Depolarized Mitochondria and Disturbances in Calcium Handling in ALS PBMCs

**Fig. 1:**
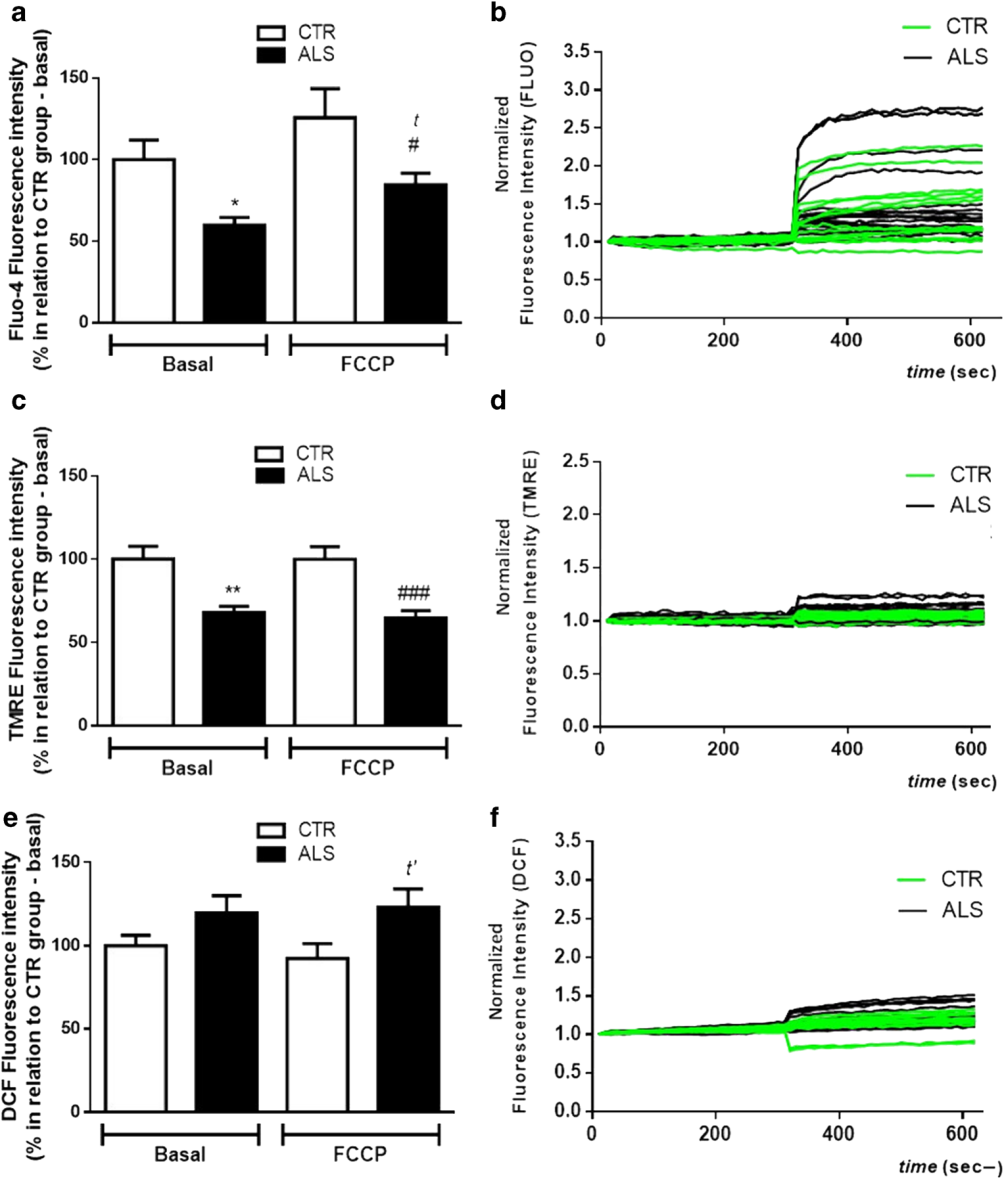
Mitochondrial depolarization and changes in calcium handling in PBMCs from ALS patients. PBMCs (300,000 cells/well—in a 96-well plate) were loaded with different fluorescent probes for 1 h at 37 °C before mitochondrial evaluation. **a** The histogram represents Fluo-4 fluorescence (10 μM) before and after stimulation with FCCP (5 μM). **b** Representative graphic line of normalized Fluo-4 fluorescence intensity in CTR/control (green lines) and ALS (black lines) samples. **c** Representative histogram of TMRE fluorescence (500 nM) before and after stimulation with FCCP (5 μM). **d** Representative graphic line of normalized TMRE fluorescence intensity of different samples in CTR/control (green lines) and ALS (black lines) samples. **e** The histogram represents the level of H_2_DCF-DA fluorescence (20 μM) before and after stimulation with FCCP (5 μM). **f** Representative graphic line of DCF normalized fluorescence intensity (values normalized to 1) in CTR/control (green lines) and ALS (black lines) samples. Data is represented by mean ± SD, and the results were normalized as a percentage of CTR/control group (basal fluorescence) (ALS group: *N* = 11; control group: *N* = 10, in duplicate). Statistical analysis was performed using one-way ANOVA followed by the post hoc Bonferroni and Student’s *t* test. It was considered significant when *p* < 0.05; *p < 0.05 and **p < 0.01, in relation control/CTR group (basal fluorescence); ^*t*^*p* < 0.05, in relation to ALS group (basal fluorescence); ^*t’*^*p* < 0.05, ^#^*p* < 0.05, and ^###^*p* < 0.001, in relation control/CTR group in the presence of FCCP

Considering the importance of mitochondria function in keeping calcium handling, and because modifications in the transport of electron and the proton motive force can interfere with ATP production [73, 74], we evaluated the cytosolic Ca^2+^ level in PBMCs from ALS and control subjects (Fig. 1a, b). As we can notice, there is a significant reduction in the Fluo-4 fluorescence in the ALS group, suggesting a decrease in cytosolic calcium in the patient’s cells. Curiously, in the presence of FCCP (5 μM), there is also a diminishment in the cytosolic calcium in ALS PBMCs.

As mitochondrial calcium buffering is influenced by ΔΨm (and vice versa), PBMCs were also loaded with TMRE. As shown in Fig. 1c, d, PBMCs from ALS patients have a significantly lower TMRE fluorescence than the control group (less TMRE in the cytosol). After the stimulation with FCCP, TMRE signal is also significantly decreased in ALS PBMCs. This data suggests that mitochondria from ALS patients are depolarized and, therefore, present less TMRE (and calcium) retention capability.

Since mitochondria are one of ROS’s primary sources, we evaluated the oxidative stress level in PBMCs from both ALS and control groups (Fig. 1e, f). Although no change is observed in basal DCF fluorescence between ALS and control PBMCs, there is a significant augmentation in DCF fluorescence after FCCP in ALS PBMCs when compared with controls’ cells.

**Fig. 2:**
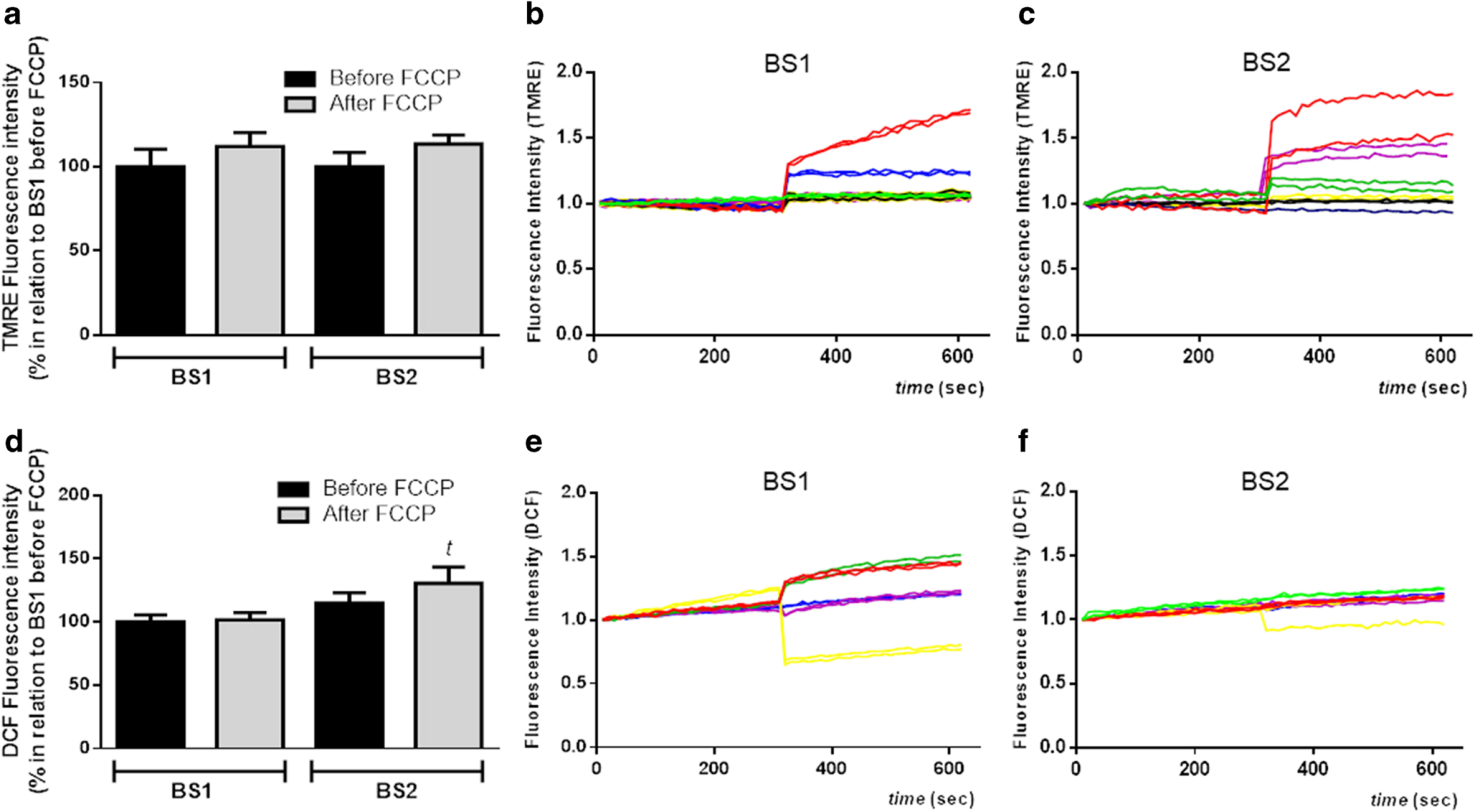
Changes in mitochondrial membrane potential and oxidative stress level in PBMCs from ALS subjects after first blood sampling (BS1) and 3 months later (BS2). PBMCs (300,000 cells/well—in a 96-well plate) were loaded with **a**–**c** TMRE (500 nM) and **d**–**f** DCF (20 μM) for 1 h at 37 °C. **a** Representative histogram of TMRE fluorescence before and after stimulation with FCCP (5 μM). **b** Representative graphic line of BS1 normalized TMRE fluorescence intensity. **c** Representative graphic line of BS2 normalized TMRE fluorescence intensity. **d** Representative histogram of DCF fluorescence before and after stimulation with FCCP (5 μM). **e** Representative graphic line of BS1 normalized DCF fluorescence intensity (values normalized to one). **f** Representative graphic line of BS2 normalized DCF fluorescence intensity (values normalized to one) (patient 1 blue, patient 2 pink, patient 3 red, patient 4 yellow, patient 5 green, patient 6 black). Data is represented by mean ± SD, and the results were normalized as a percentage of BS1 group before FCCP (basal) (*N* = 6, in duplicate). Statistical analysis was performed using one-way ANOVA followed by the post hoc Bonferroni and Student’s *t* test. It was considered significant when *p* < 0.05; ^*t*^*p* < 0.05, in relation to BS1 after FCCP

To test if ALS patients present changes in mitochondrial functional parameters with time, named mitochondrial membrane potential and oxidative stress, samples were re-evaluated in 6 patients after 3 months of the first blood sampling (BS1). As shown in Fig. 2a, c, there is no difference regarding TMRE fluorescence between groups (BS1 and BS2), neither before nor after FCCP. Although we do not observe any change in basal DCF fluorescence with time, the DCF signal is significantly higher in the ALS group in BS2 in comparison with that in BS1 after FCCP (Fig. 2d–f).

### Decreased Antioxidant Defense and Metabolic Gene Expression in ALS PBMCs

**Fig. 3:**
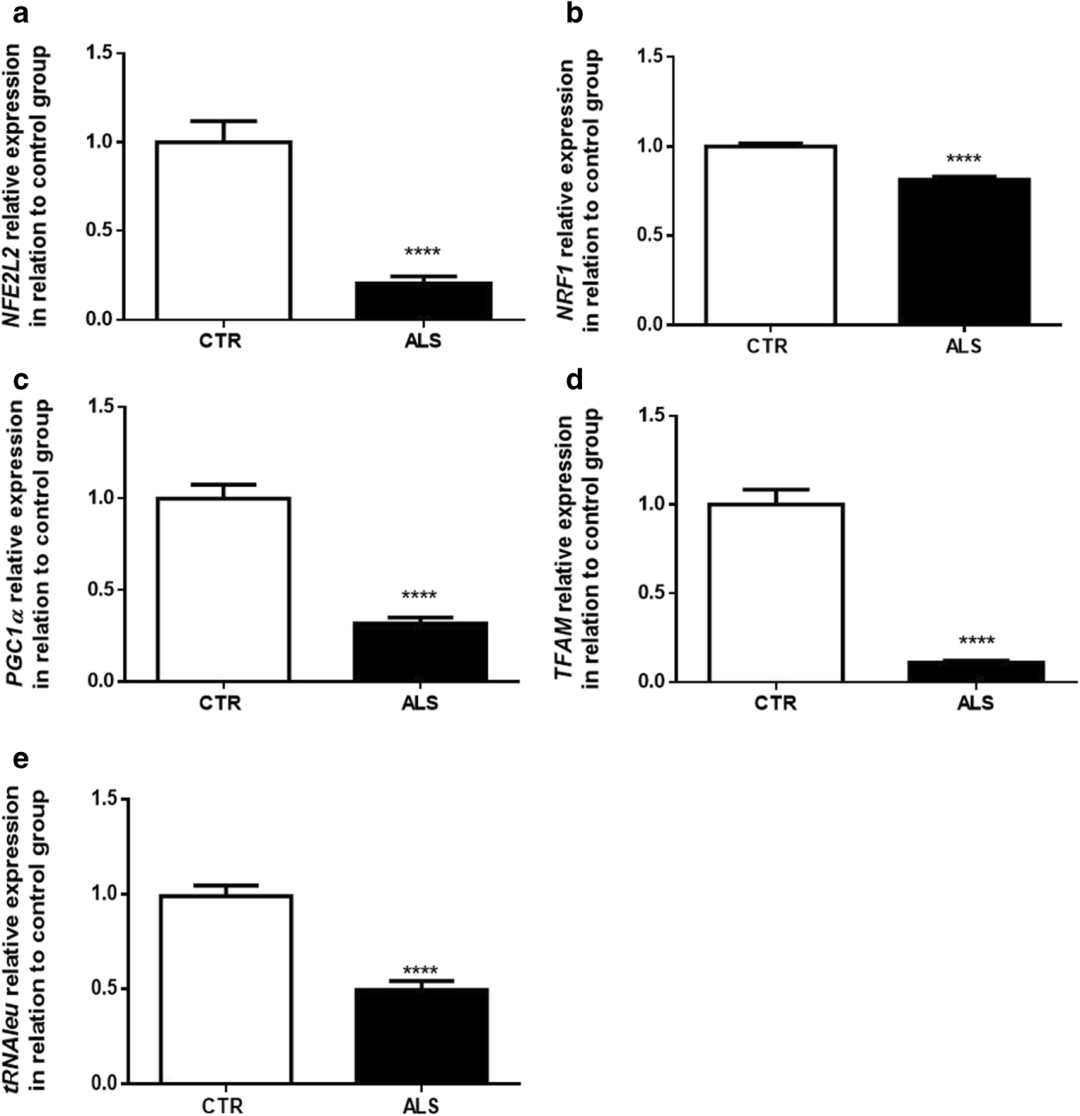
Diminishment in the expression of genes related to mitochondrial metabolism, biogenesis, and content in ALS PBMCs. Relative expression (2^−ΔΔCT^) of *NFE2L2* (**a**), *NRF1* (**b**), *PGC-1α* (**c**), *TFAM* (**d**), and *tRNAleu* (**e**) in relation to actin and control group. Data are represented by mean ± SD (*N* = 5, in duplicates) and the results expressed in 2^−ΔΔCT^. Statistical analysis was performed using Student’s *t* test. It was considered significant when *p* < 0.05; ****p < 0.0001, in relation to CTR/control group

Because oxidative stress is increased in peripheral cells from ALS subjects, we further investigated the expression of *NFE2L2*, a transcription factor related to the synthesis of antioxidant enzymes [57, 75–77]. As shown in Fig. 3a, there is a massive reduction in *NFE2L2* in ALS cells, indicating that ALS PBMCs have a decreased antioxidant defense [78–80].

Regarding the expression *NRF1*, responsible for regulating several nuclear-encoded electron transport chain proteins [70, 81, 82], there is a significant diminishment in ALS cells compared with taht in controls (Fig. 3b).

### Reduced Mitochondrial Biogenesis and Content in PBMCs from ALS Patients

To evaluate changes in mitochondrial biogenesis and content, we assessed *PGC-1α*, *TFAM*, and *tRNAleu* expression in PBMCs [59, 65, 66, 68, 83–92]. As shown in Fig. 3c, there is a significant decrease in *PGC-1α* expression in ALS cells. Corroborating this data, we demonstrated that ALS PBMCs also present a significant decrease in *TFAM* and *tRNAleu* expression (Fig. 3d, e).

### Increased Mitochondrial Fission and Autophagy Gene Expression in ALS Patients’ Cells

**Fig. 4:**
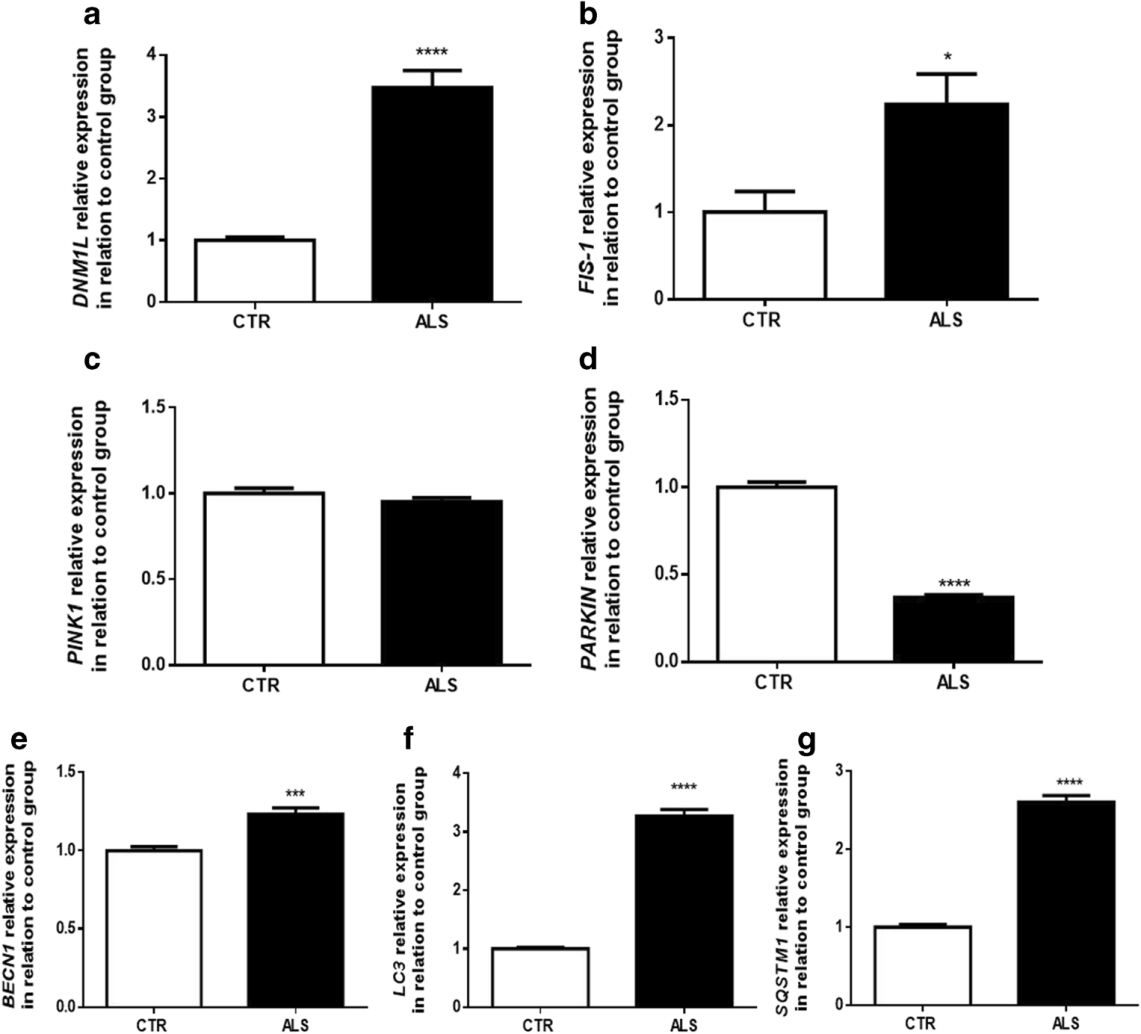
Increase in mitochondrial fission genes expression and diminishment in the expression of mitochondrial degradation genes, despite an augmentation in autophagy, in PMBCs from ALS patients. Relative expression of *DNM1L* (**a**) and *FIS-1* (**b**) (genes linked to mitochondrial fission), *PINK* (**c**) and *PARKIN* (**d**) (genes related to mitochondrial target degradation through autophagy), and *BECLIN* (**e**), *LC3* (**f**), and *SQSTM1* (**g**) (genes of autophagy pathway) in relation to actin and control group. Data in graphs are the mean ± SD (*N* = 5, in duplicates) and the results expressed in 2^−ΔΔCT^. Statistical analysis was performed using Student’s *t* test. It was considered significant when *p* < 0.05; *p < 0.05, ***p < 0.001, ****p < 0.0001, in relation to CTR/control group

Knowing that mitochondrial membrane potential and biogenesis are related to modifications in mitochondrial dynamics [93, 94], we investigated the expression of *DNM1L* and *FIS-1* in PBMCs; both genes are linked to mitochondrial fission [95–97]. We observe a significant enrichment in both gene expressions in ALS patients’ PBMCs compared with that in the controls, indicating an augmentation in organelle fission (Fig. 4a, b).

In order to understand if the augmentation in fission machinery could lead to an increase in mitophagy [98, 99], we analyzed the expression of both *PINK1* (responsible to preserve of mitochondrial functioning and integrity) and *PARKIN* (recruited by PINK1 for the degradation of damaged mitochondria) [100, 101]. There is no significant difference in the expression of *PINK1* between ALS and control groups (Fig. 4c). However, the expression of *PARKIN* is significantly lower in PBMCs from ALS patients (Fig. 4d). This outcome indicates that despite mitochondrial function is deregulated, fewer mitochondria seems to be degraded.

To evaluate whether ALS PBMCs present changes in autophagy-related gene expression, we examined *BECN1* (essential for nucleation/expansion of autophagosome), *LC3* (responsible for autophagosome membrane formation), and *SQSTM1* (in charge of the recruitment of specific cargo) [102–107]. It is possible to observe that there is a significant increment in the expression of all genes tested in ALS patients’ cells when compared with controls’ PBMCs (Fig. 4e–g). This data indicates that despite lower levels of *PARKIN* in ALS patients’ PBMCs, the autophagy pathway seems to be activated.

### Changes in Bioenergetics Metabolic Compounds in PMBCs from ALS Patients

**Fig. 5:**
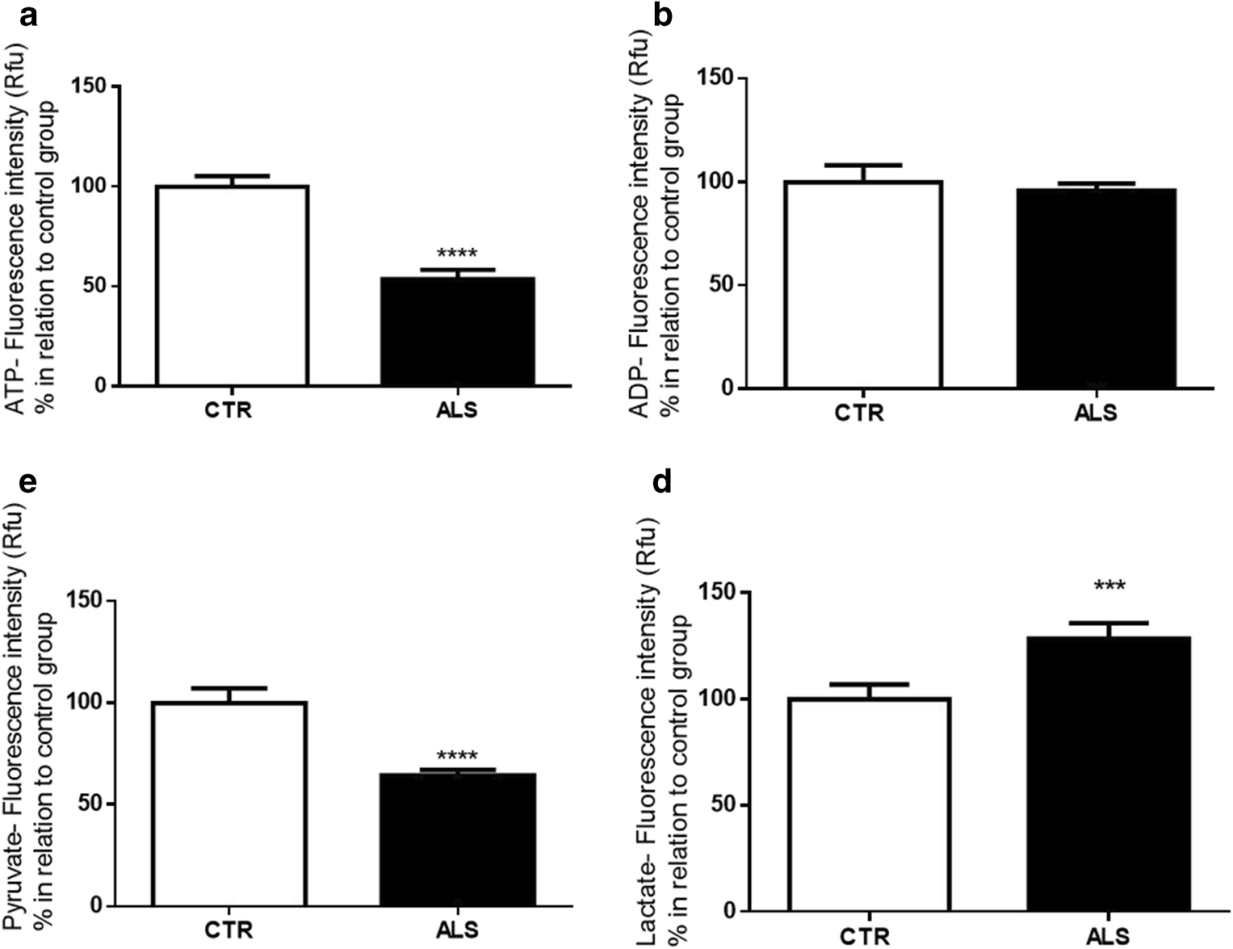
Changes in bioenergetics metabolic compounds (ATP, ADP, pyruvate, lactate) in PMBCs from ALS patients. **a** ATP, **b** ADP, **c** pyruvate, and **d** lactate levels. Data in graphs are the mean ± SD (*N* = 3, in duplicates), and the results were normalized as a percentage of the control group. Statistical analysis was performed using Student’s *t* test. It was considered significant when *p* < 0.05; ***p < 0.001 and ****p < 0.0001, in relation to CTR/control group

**Fig. 6:**
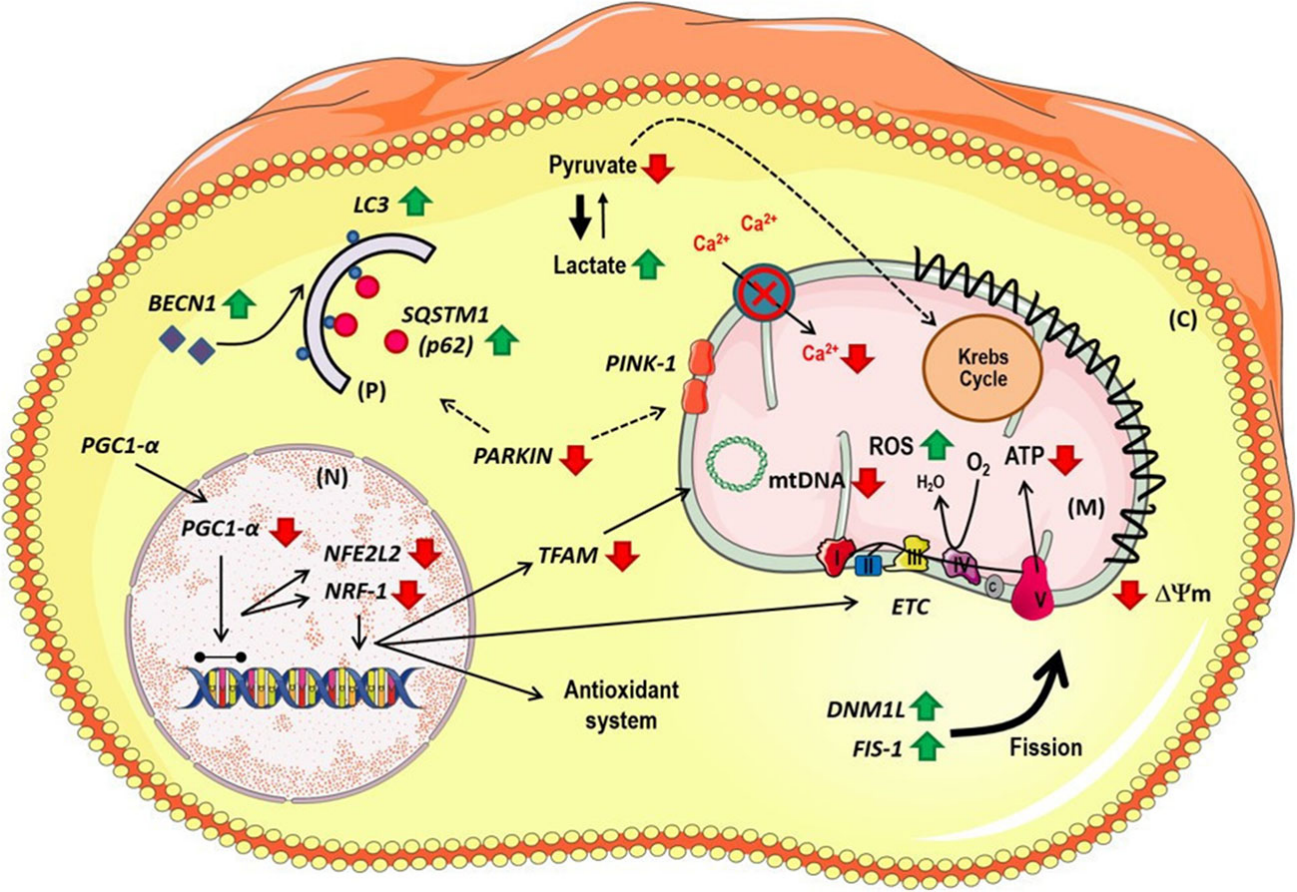
Schematic representation of mitochondrial deregulation observed in peripheral blood mononuclear cells from ALS patients. Cells from ALS patients present mitochondrial depolarization and diminishment in mitochondrial calcium uptake and/or retention, changes in redox homeostasis, and a decrease in metabolism biogenesis–related gene expression, namely, *PGC-1α*, *NFE2L*, *NRF1*, *TFAM*, and *tRNAleu*. Moreover, PBMCs from ALS individuals show an augmentation in mitochondrial fission (represented by an increase in *DNM1L* and *FIS-1* expression) and a reduction in mitochondrial degradation signaling since there is a significant decrease in *PARKIN* expression. Notwithstanding, there is a significant increase in autophagy-related genes, as *BECLIN*, *LC3*, and *SQSTM1*. All these changes can further contribute to the decreased levels of ATP production and pyruvate in ALS PBMCs, and the augmented lactate levels observed in patients’ cells

As we observed changes in mitochondrial function in ALS’ PBMCs, besides deviations in the expression of genes related to mitochondrial metabolism, biogenesis, and fission, we investigated whether the bioproducts related to energy metabolisms, such as ATP, ADP, pyruvate, and lactate, could vary. As observed in Fig. 5a, there is a significant decrease in ATP levels in PBMCs from ALS patients compared with control individuals; no modification is observed regarding ADP levels (Fig. 5b). As we can notice in Fig. 5c, the pyruvate level significantly dropped in ALS PBMCs, while the level of lactate is significantly elevated (Fig. 5d). This outcome suggests that pyruvate may be converted into lactate to produce energy since ATP synthesis, once oxidative phosphorylation might be jeopardized because of mitochondrial deregulation (Fig. 6).

## Discussion

In this manuscript, we showed that PBMCs from ALS patients present a lower mitochondrial calcium uptake/retention, mitochondrial depolarization, and redox homeostasis unbalance, together with a significant decrease in vital metabolic gene expression and a diminishment in mitochondrial biogenesis and content. These outcomes seem to occur in parallel with an increase in mitochondrial fission and autophagy-related gene expression, despite a reduction in mitochondrial degradation signaling demonstrated by *PINK* and *PARKIN* expression. Accordingly, ALS cells present a decrease in high energy–producing metabolic compounds, corroborating the fact that mitochondrial deregulation can lead to an energetic deficiency in peripheral cells as an ultimate consequence of the disorder. Thus, mitochondrial function evaluation in PBMCs could be a valuable strategy to detect ALS, as well as to assess its progression and therapy efficacy.

An intensification in mitochondrial calcium influx is described as the primary process linked to cell death and excitotoxicity [108–113]. Hence, we evaluated calcium homeostasis in PBMCs from control and ALS subjects. As we show in Fig. 1a, there is a significant decrease in the basal fluorescence of Fluo-4 in the ALS group, along with a lower fluorescence in ALS PBMCs after stimulation with FCCP. Such data is in agreement with the literature and indicates that probably less mitochondria are buffering the ion, which could lead to cellular dysfunction [114–116].

Because calcium homeostasis is necessary for keeping the mitochondrial membrane potential [117, 118], and mitochondria membrane potential plays a direct role in calcium handling [119], we indirectly evaluated such parameters using the fluorophore TMRE [120]. As shown in Fig. 1c, there is a significant decrease in the basal TMRE fluorescence in patients’ cells. Because TMRE is a cationic indicator (accumulates in hyperpolarized mitochondria), we hypothesized that if mitochondria are depolarized, as indicated by Fluo-4 experiments, TMRE fluorescence would not vary in the presence of FCCP (mitochondria would not internalize TMRE). Indeed, as we conjectured, after FCCP, TMRE fluorescence was still significantly lower in the ALS group when compared with that in the control. Similarly, other studies also showed a decrease in ΔΨm [47, 121, 122]. Thus, the decreased capability of ALS PBMC mitochondria in uptake and/or retaining calcium might be related to the organelle’s depolarization. These results reinforce the need to identify biomarkers using both blood samples and mitochondrial functional parameters as a target.

As calcium homeostasis and ΔΨm interfere with the electron transport chain and, therefore, oxidative stress levels [123, 124], we investigated general ROS formation in PBMCs. Indeed, physiologically, mitochondria are one of ROS’s major sources, together with several mechanisms, but ROS are a key factor in the etiology of various neurodegenerative pathologies since its levels are associated with neuronal degeneration [36, 125–129]. The results demonstrate that there is more ROS in ALS PBMCs than in the control group in both basal (although not significant) and post-FCCP conditions (Fig. 1e). Such findings might be due to an impaired antioxidant system. Indeed, it was already demonstrated a significant diminishment in antioxidant defense markers in patients with ALS, including reduced glutathione in the motor cortex of ALS patients. Nonetheless, the results are somehow inconsistent due to the heterogeneity of patients [129]. Importantly, our data is in accordance with literature that also shows a small increase in oxidative stress levels in ALS PBMCs at the baseline and a significant increase after FCCP [130–132]. Moreover, and corroborating our findings, a clinical study using serum and whole blood from 10 ALS patients demonstrated an increment in malondialdehyde and 8-hydroxy-2′-deoxyguanosine levels [36].

Since ALS is a progressive degenerative disorder, we also investigated whether PBMCs from ALS patients present modification in mitochondrial functional parameters with time. Unfortunately, the amount of blood in the BS2 was lower than in the BS1, a reflection of the fragile patients’ veins. Consequently, we evaluated membrane potential (Fig. 2a) and oxidative stress level (Fig. 2b). As shown, only the DCF signal, after the addition of FCCP, is significantly higher in the BS2 group in relation to the BS1. This result suggests that patients become more susceptible with time to toxic stimuli, such as FCCP. The increase in the fluorescence could be due to a slight decrease in basal DCF fluorescence in CTR group, an impaired antioxidant system, and a further leak of electron from the mitochondrial complexes chain due to the stimulation of electron’s transport through the mitochondrial complexes chain induced by FCCP. It is also important to mention that DCF is capable of providing data about general ROS synthesis, and no specific measurements of ROS were performed (superoxide, hydrogen peroxide, MDA, or HNE). For this reason, more studies are necessary to investigate whether other mitochondrial parameters related to oxidative stress modulation change with ALS progression. It is essential to mention that the lower number of patients reassessed was due to patients missing scheduled medical appointments because of motor difficulties. Although only six patients were re-evaluated, and the period between BSs was 3 months, we did observe a significant increase in oxidative stress.

Due to the increased ROS level in ALS PBMCs, we decided to investigate *NFE2L2* expression [57], which is implicated in cellular detoxifying systems [78–80]. We observed a large diminishment in *NFE2L2* expression (more than 50%) in ALS cells (Fig. 3a). The increased levels of ROS could have been related to a failure of *NFE2L2* in translocating to the nucleus, reducing the transcription of secondary factors of the antioxidant cascade [79, 133]. Interestingly, in previous work with PBMCs, the expression of *NFE2L2* had no changes in relation to controls, but ALS patients were 10 years older than in our study [13], which could indicate a time-dependent *NFE2L2* expression. In agreement with our outcome, the expression of *NFE2L2* was declined in humans with ALS and in the SOD1-G93A model [134, 135]. Of importance, *NFE2L2* overexpression was associated with more prolonged survival and neuroprotection in the SOD1-G93A mouse model [134, 136].

Considering that mitochondrial function can vary due to changes in mitochondrial protein content, including in mitochondrial complexes, we further investigated the expression of *NRF1*. Our study demonstrated a significant decrease in *NRF1* expression in ALS PBMCs (Fig. 3b), a finding that corroborates previous data in the literature in which a reduction in *NRF1* expression in the spinal cord and muscles from ALS patients and SOD1-G93A animal models was shown [135, 137]. Our data then suggest that mitochondria from ALS PBMCs can present less mitochondrial complex inhibiting, consequently, mitochondrial oxidative phosphorylation system (OXPHOS).

Given that there is a significant reduction in both *NFE2L2* and *NRF1* expressions in PBMCs from ALS patients, and that *PGC-1α* regulates both, we investigated mitochondrial biogenesis in our cellular model. Importantly, *PGC-1α* leads to an increase in mitochondrial mass and number, stimulating the division of pre-existing mitochondria in order to sustain energetic cellular status [55, 138]. As we can observe in Fig. 3c, there is a significant decrease in *PGC-1α* expression in the ALS group. This evidence aligns with a diminished expression of *PGC-1α* in the spinal cord of SOD1-G93A mice [139], and in muscle and motor cortex of human sporadic ALS [135]. Intriguingly, low *PGC-1α* expression is related to lower mitochondrial activity and to a worsened regulation of reactive oxygen species [54, 55], and fewer *PGC-1α* seems to be associated with a decrease in mitochondrial activity [140] and an increase in muscle degeneration in a different animal model of ALS [141, 142]. Therefore, decreasing *PGC-1α* may contribute to minor ROS protection [143–145]. These findings seem to corroborate our data so far and can also be associated with changes in the basal level of cytosolic Fluo-4 and TMRE.

To corroborate the reduction in mitochondrial biogenesis, we also evaluated *TFAM* expression, a mitochondrial transcription factor regulated by *PGC-1α* responsible for the transcription of mtDNA genes and bioenergetic function [146–148]. We detected significant mitigation in *TFAM* expression in the ALS group (Fig. 3d). Curiously, a drop in *TFAM* expression was associated with an augmentation of oxidative damage [149]. In line with our data, a reduction in *TFAM* was also observed in the spinal cord and motor cortex of human sporadic ALS [135]. We also observed a decrease in *tRNAleu* expression (Fig. 3e), a unique sequence in mtDNA, suggesting fewer copies of mtDNA in ALS cells [150]. A reduced mtDNA copy number was also described on neurons and the spinal cords of ALS patients [148, 151]. Therefore, even with a limited sample size, we do consider that our results represent a true argument in favor of an augmentation in mitochondrial biogenesis, as four different approaches were assessed.

Cellular death among degenerative disorders is commonly associated with an increase of mitochondrial fission [152]. Indeed, fission can decrease energy production, encourage oxidative stress, lead to mtDNA deletion, and impair calcium buffering [153]. Remarkably, mitochondrial fission can be stimulated by oxidative stress [154–156]. Because we observed a diminishment in mitochondrial biogenesis and in mtDNA copy number, as well as changes in calcium homeostasis, we also evaluated *DNM1L* and *FIS-1* expression in PBMCs from ALS and control groups. As shown in Fig. 4a, b, there is a significant increase in the expression of both genes in patients’ cells. Significantly, and corroborating our findings, an increase in fission, including changes in DNM1L and FIS-1*,* was already reported in neurons from fALS patients [157, 158], in ALS lymphoblastoid human cells [159], in ALS patient–derived fibroblasts [96], in cultured motor neurons expressing SOD1 mutant [96], and in animal models of ALS [154, 157, 160]. Thus, so far, our data implies that mitochondrial functional parameters and dynamics should be further investigated as a biomarker, even though we had few samples and no further techniques to explore such processes, namely, Western blot or immunofluorescence.

Taking into consideration that changes in *DNM1L* and *FIS-1* expression, together with mitochondrial depolarization and biogenesis modification, are related to selective mitochondrial degradation [56, 161], we hypothesized that mitophagy, the major machinery to eliminate dysfunctional mitochondria [162], could also be dysfunctional in PBMCs from ALS subjects. Subsequently, we investigated in PBMCs the expression of *PINK-1* and *PARKIN*, genes related to mitophagy [99, 163–165]. It is important to point out, however, that although PARKIN is associated with mitophagy [166], its translocation to deregulated mitochondria is one of the earlier steps in mitochondrial control quality [167]. As shown in Fig. 4c, there is no difference in *PINK-1* between groups, but there is a significant decrease in *PARKIN* in ALS PBMCs. Supporting our results, Lagier-Tourenne, Stribl, and their colleagues also observed a diminishment in *PARKIN* in samples from autopsied brains of ALS patients and from hTDP-43A315T animals [168, 169]. Therefore, our data suggest that the ubiquitination of target proteins enrolled in autophagy machinery could be impaired in PBMCs from individuals with ALS.

To investigate whether autophagy genes would be changed in PBMCs from ALS patients, we verified the expression of *BECN1*, *LC3*, and *SQSTM1* (Fig. 4e–g). We showed a significant augmentation in all three genes in ALS cells. In this sense, an increase in autophagy-related gene expression could be a compensatory mechanism in an attempt to clear out unwanted organelles. Our data is supported by the literature, in which transgenic models have increased *BECN1*, *LC3*, and *SQSTM1* expression [170–175]. It should be noted, still, that in respect to autophagy, our goal in verifying *BECN1*, *LC3*, and *SQSTM1* expressions was to identify a possible alteration in this mechanism (in general), rather than examine the functionality/blockage of the whole pathway (initiation, formation, and maturation). To investigate autophagy in greater depth, various methodologies including fluorescence and high-content imaging, flow cytometry, and luminescence detection could be performed [102, 107]. Therefore, future studies should also look for autophagy markers (end-points) in ALS cohorts.

Since we observed changes in organelles’ functional parameters, specifically calcium handling, membrane potential, and oxidative stress level, together with modification in the expression of genes related to mitochondrial dynamics and degradation in ALS cells, we additionally evaluated the level of energy metabolites (Fig. 5). Besides the decreased ATP levels observed in PBMCs from ALS patients (Fig. 5a), there was also a reduction in the pyruvate status (Fig. 5c) and an increased level of lactate (Fig. 5d). Our findings seem to be in accordance with the literature that shows a diminishment in ATP levels [176–178], denoting that mitochondrial deregulation is leading to a decrease in energy reservoir. Moreover, a reduced level of pyruvate was demonstrated in SOD1-G93A mice [179, 180], as well as an increment in lactate in the same rodent model, in ALS homogenate from sporadic patients, and in G39-neuroblastoma spinal cord mutant cells [181–183]. The lactate outcomes are consistent with an augmentation in the aerobic glycolysis, the so-called Warburg effect that has been noted in ALS [184]. In fact, lactate augmentation accounts for a compensatory glycolytic response to favor ATP synthesis [185]. Although the synthesis of ATP through lactate is not efficient and is less cost-effective than through OXPHOS, metabolic modifications might be an attempt to restore energy homeostasis [186] since ATP depletion contributes to ALS progression [187]. Furthermore, the accumulation of lactate can be a strategy to convert NADH (reduced form of nicotinamide adenine dinucleotide) into NAD+ (oxidized form of nicotinamide adenine dinucleotide), reverting reductive stress in order to compensate for an oxidative phosphorylation deficiency [186, 188].

To date, the most common medication used by ALS patients is Rilutek^TM^ (riluzole) [189–191], known as a wide-spectrum medication [9, 39, 192–195] and showing different effects in later stages of the disease [192, 196]. Therefore, one might think that the data presented would point toward the effect of riluzole. However, it is important to mention that our findings are in opposition to riluzole’s influence described so far [192, 196]. Thus, it can be assumed that at least in part, the results presented herein are not riluzole-dependent.

Altogether, this work brings new insights into how ALS affects peripheral blood cells and how mitochondria deregulation plays a role in this hallmark. Specifically, it seems that PBMC mitochondria from ALS patients become depolarized, losing its ability to uptake and/or retain calcium from cytosol, contributing to the increase in oxidative stress and decrease in *NFE2L2* expression. ALS PBMCs also present lower mitochondrial content, which may further corroborate the decreased cytosolic levels of Fluo-4 and TMRE observed. Notably, as mentioned, despite fewer mitochondria, the ROS level is slightly higher in ALS cells. Moreover, ALS PBMCs present an unblemished decrease in metabolic intermediates, indicating that, ultimately, the energetic cellular homeostasis is jeopardized. Importantly, the collapse of mitochondrial membrane potential, indicated by TMRE fluorescence, and high levels of calcium and ROS are triggered by mitochondrial degradation through mitophagy. Oddly, ALS PBMCs present a decrease in *PARKIN*, which is needed to target malfunctioning mitochondria and recruit autophagy proteins. Eventually, the accumulation of dysfunctional organelles could contribute to a worsening ALS scenario.

It should be noted that our sample size is small, as several subjects in the Neurology Department presented motor neuron disorders that were not classified as ALS. Nevertheless, not only should the sample size be increased in future studies but other time points could also be checked. Although the outcomes presented here seem not to be riluzole-dependent, additional studies should be carried out in order to investigate these biases. Moreover, the lack of knowledge regarding secondary mechanisms related to the disease progression represents a struggle for the academic community, especially because diagnosis and prognosis are mainly based on physical exams [23]. Unfortunately, this is a matter of debate that affects all studies performed with ALS patients [9].

It is important to emphasize, though, that we established a parallel between the outcomes presented with findings in (i) ALS animal models, (ii) ALS lymphocytes (established culture bought from cell banks), (iii) neuroblastomas expressing mutant proteins, (iv) iPSC derived-neurons from ALS patients with specific mutations, and (v) human brain samples. Therefore, the novelty of our work is as follows. (1) At once, we could show changes observed in a variety of ALS models and human tissues in the same ALS patients’ cell type, PBMCs. (2) ALS patients from our study were 93% sALS subjects. (3) Mitochondrial functional parameters and gene expression evaluated in our work seem not to be correlated with the diagnosis of fALS—results from the only one fALS individual were under the ALS group’s mean. (4) All the parameters showed were measured in only 8 mL of blood (sampled once). (5) It was used an only primary culture of PBMCs from ALS patients along with the study, and no cellular transformation was performed in these cells.

Curiously, in the literature, very few studies reassess cellular mechanisms in ALS patients’ samples along with the progression of the disorder. To date, to our knowledge, these works focus on clinical evaluation, epidemiology, oximetry, respiratory failure, and genetic counseling. Hence, we can say that we are one of the groups that considered this fact; we could match our results regarding *NFE2L2* expression with patients 10 years older than those in our group. Such finding is auspicious, since the values differ, showing a change in this transcription factor over time. Considering that we evaluated distinct parameters investigated in many models and human samples and that such changes are observed in fALS and sALS, the assessed factors might be a significant biomarker to study disease progression in both familial and sporadic ALS patients.

## Conclusion

Overall, our results indicate that PBMCs from ALS patients present a significant mitochondrial dysfunction that could be used as a powerful tool in investigating ALS disclosure and improvement. Importantly, the usage of PBMCs in biomarker research was already validated, and our findings can further contribute to future findings concerning new pharmacological strategies against ALS.

## Electronic Supplementary Material

ESM 1 (PDF 286 kb)

## Data Availability

All data generated or analyzed during this study are included in the manuscript and the supplementary files.

## Abbreviations

ΔΨm: Mitochondrial membrane potential
ALS: Amyotrophic lateral sclerosis
ADP: Adenosine diphosphate
ATP: Adenosine triphosphate
BECN1: Beclin 1
BS1: Blood sampling 1
BS2: Blood sampling 2
CaCl_2_: Calcium chloride
CM-H2DCF-DA: Carboxylated diclohydro-fluorescein reagent
DNM1L: Dynamin-related protein 1
fALS: Familial cases of ALS
FCCP: Fluorocarbonyl cyanide phenylhydrazone
FIS-1: Mitochondrial fission 1
FTD: Frontotemporal dementia
iPSC: Induced pluripotent stem cells
ISCMSP: Irmandade da Santa Casa de Misericórdia de São Paulo
LC3: Microtubule-associated proteins 1A/1B light chain 3
Min: Minutes
mRNA: Messenger RNA
mtDNA: Mitochondrial DNA
NAD+: Oxidized form of nicotinamide adenine dinucleotide
NADH: Reduced form of nicotinamide adenine dinucleotide
NFE2L2: Nuclear factor (erythroid-derived2)-like 2
NRF1: Nuclear respiratory factor 1
PARKIN: E3 ubiquitin-protein ligase parkin
PBMCs: Peripheral blood mononuclear cells
PDH: Pyruvate dehydrogenase complex
PGC-1α: Peroxisome proliferator-activated receptor gamma coactivator 1-alpha
PINK1: PTEN-induced putative kinase protein 1
qRT-PCR: Real-time PCR
ROS: Reactive oxygen species
sALS: Sporadic cases of ALS
SD: Standard deviation
SQSTM1: Sequestosome-1
TFAM: Mitochondrial transcription factor A
TMRE: Tetramethylhydrodamine ethyl ester
Δ: Difference between the fluorescence after FCCP and the basal fluorescence intensity
