# Supplementary material for: Decreased Mitochondrial Function, Biogenesis, and Degradation in Peripheral Blood Mononuclear Cells from Amyotrophic Lateral Sclerosis Patients as a Potential Tool for Biomarker Research
Source: Mol Neurobiol. 2020 Aug 25;57(12):5084–102. doi: 10.1007/s12035-020-02059-1 (PMC7541388; doi:10.1007/s12035-020-02059-1)
Supplement: Supplementary file 1 — (PDF 286 kb) [file 12035_2020_2059_MOESM1_ESM.pdf]

SUPPLEMENTARY MATERIAL

Decreased mitochondrial function, biogenesis and degradation in peripheral blood mononuclear cells from Amyotrophic Lateral Sclerosis patients as potential tool for biomarker research

Beatriz Grisolia Araujo[1], Luiz Felipe Sousa e Silva[1], Jorge Luiz de Barros Torresi[1], Amanda Siena[1], Berenice Cataldo Oliveira Valerio[2], Mariana Dutra Brito[1], Tatiana Rosado Rosenstock[1]*

1. Department of Physiological Science, Santa Casa de São Paulo School of Medical Science, São Paulo/SP, Brazil; 2. Department of Neurology, Irmandade da Santa Casa de Misericórdia de São Paulo, São Paulo/SP, Brazil

*Corresponding author:
Tatiana Rosado Rosenstock
Rua Doutor Cesário Motta Júnior, 61 - Vila Buarque
São Paulo, SP, Brazil
CEP 01221-020
E-mail: tati.farm@gmail.com; tatiana.rosado@fcmsantacasasp.edu.br

Material and Methods

PBMCs viability

To verify the viability of PBMCs used in our studies, cells were stained with Trypan Blue (1:1); Trypan Blue is a tetrasulfonated anionic dye that only permeates the cell if the membrane is disrupted (non-viable cell indicator). Thus, after homogeneizing cells with Trypan Blue, viable (translucent) cells were counted manually. The number of viable cells were calculated per mL and data were plotted as a percentage of the control group.

Gene expression

Mitochondrial function was also evaluated by real-time PCR (qPCR) in 7500 Real-Time PCR Instrument. Specifically, we investigated the expression of the following genes: i) Nuclear respiratory factor 1 (NRF1), ii) Nuclear factor (erythroid-derived2)-like 2 (NFE2L2), iii) Peroxisome proliferator-activated receptor gamma coactivator 1-alpha (PGC-1α), iv) mitochondrial transcription factor A (TFAM), v) tRNAleu, vi) Dynamin related protein 1 (DNM1L), vii) Mitochondrial fission 1 (FIS-1), viii) PTEN-induced putative kinase protein 1 (PINK1), ix) E3 ubiquitin-protein ligase parkin (PARKIN), x) Beclin 1 (BECN1), xi) Microtubule-associated proteins 1A/1B light chain 3 (LC3), and xii) Sequestosome-1 (SQSTM1) (Kiyama et al., 2018; Tanida et al., 2008; Rubinsztein et al., 2009; Sun et al., 2009; Matsunaga et al., 2009; Li et al., 2016; Oselamme et al., 2012; Kim et al., 2007; Copple et al., 2008; Weydt et al., 2006; Weydt et al., 2014; Choi et al., 2014; Rapaport et al., 2009; Bayer et al., 2017; Handschin, 2009; Venegas and Halberg, 2012; Handschin and Spiegelman, 2006; King et al., 2018; Bayer et al., 2017). Gene expression was determined with $2^{-\Delta\Delta CT}$ (Livak and Schmittgen, 2001). All the sequences are shown below (Table 1).

Table 1: Primers used to investigate mitochondrial metabolism, biogenesis (content), dynamic and degradation (forward and reverse sequences 5'-3').

Gene	Forward 5'-3'	Reverse 5'-3'
Nuclear factor (erythroid-derived 2)-like 2 (*NFE2L2*)	GTCCAGCAGTGCAGCTCA	CCTGTTCCTGCTCTGCTG
Nuclear respiratory factor 1 *(NRF1)*	CGAGGACACCTCTTACGAT	GAGATACAGAGGACAATAGC
Peroxisome proliferator-activated receptor gamma coactivator 1-alpha (*PGC-1α*)	CTCATTTGATGCFCTGACAG	GGTACTGAGACCACTGCAT
Mitochondrial transcription factor A (*TFAM*)	GGTCGGGTCATGCCTCAT	ACAGATGAAAACCACCTCGG
tRNAleu	CACCCAAGAACAGGGTTTGT	TGGCCATGGGTATGTTGTTA
Dynamin related protein 1 (*DNM1L*)	GCAGCTGATATGCTAAAGGC	ATAGCAGTCTCCACATGAG
Mitochondrial fission 1 *(FIS-1)*	CAGTCTGAGAAGGCAGCAG	GACGTAATCCCGCTGTTCC
PTEN-induced putative kinase protein 1 (*PINK1*)	GTCAGGAGACAAGACAGCG	CTCTCTGTGGATGCAGGC
E3 ubiquitin-protein ligase parkin (*PARKIN*)	GACGGCAGCGAATCATGTC	GTGGATTCCTTGCCGAGC
Beclin 1 (*BECN1*)	CCTGGACTGTGTGCAGCA	TTCGTCAGCATGAACTTGAG
Microtubule-associated proteins 1A/1B light chain 3 (*LC3*)	CGAGAGCAGCATCCAACC	GACCATGCTGTGTCCGTTC
Sequestosome-1 (*SQSTM1*)	GGTTCAGATAATGCCCTGG	TGTCAATTCCTCGTCACTGG

Results

Viability of ALS and control subjects PBMCs

For a cell viability check in both experimental groups, we quantified as intact (healthy) cells according to the Trypan Blue protocol. We can observe that there is a small difference between the viable cells between the two groups.

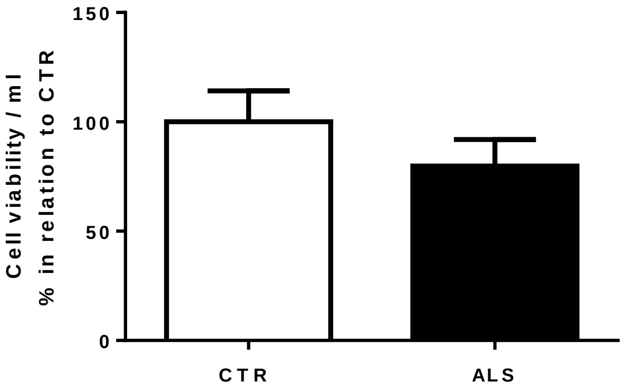

Figure 1SS: Evaluation of cellular viability (by Trypan Blue) in PBMCs of ALS patients and controls. There is no significant difference between groups by Student's t test ($p > 0.05$).
